# Supplementary material for: Specific Disruption of Ras2 CAAX Proteolysis Alters Its Localization and Function
Source: Microbiol Spectr. 2023 Jan 5;11(1):e02692-22. doi: 10.1128/spectrum.02692-22 (PMC9927470; doi:10.1128/spectrum.02692-22)
Supplement: Supplemental file 1 — Supplemental material. Download spectrum.02692-22-s0001.pdf, PDF file, 3.8 MB [file spectrum.02692-22-s0001.pdf]

Supplementary Figures:

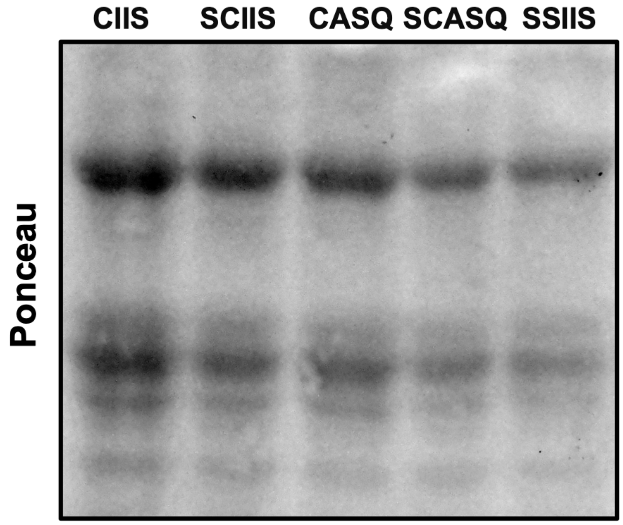

Figure S1: Confirmation of sample loading amounts. Ponceau S staining of blot used for **Fig. 3A** prior to antibody incubation. Different volumes of lysates were loaded on the gel (CIIS – 5x; SCIIS and CASQ – 2.5x; SCASQ and SSIIS – 1x).

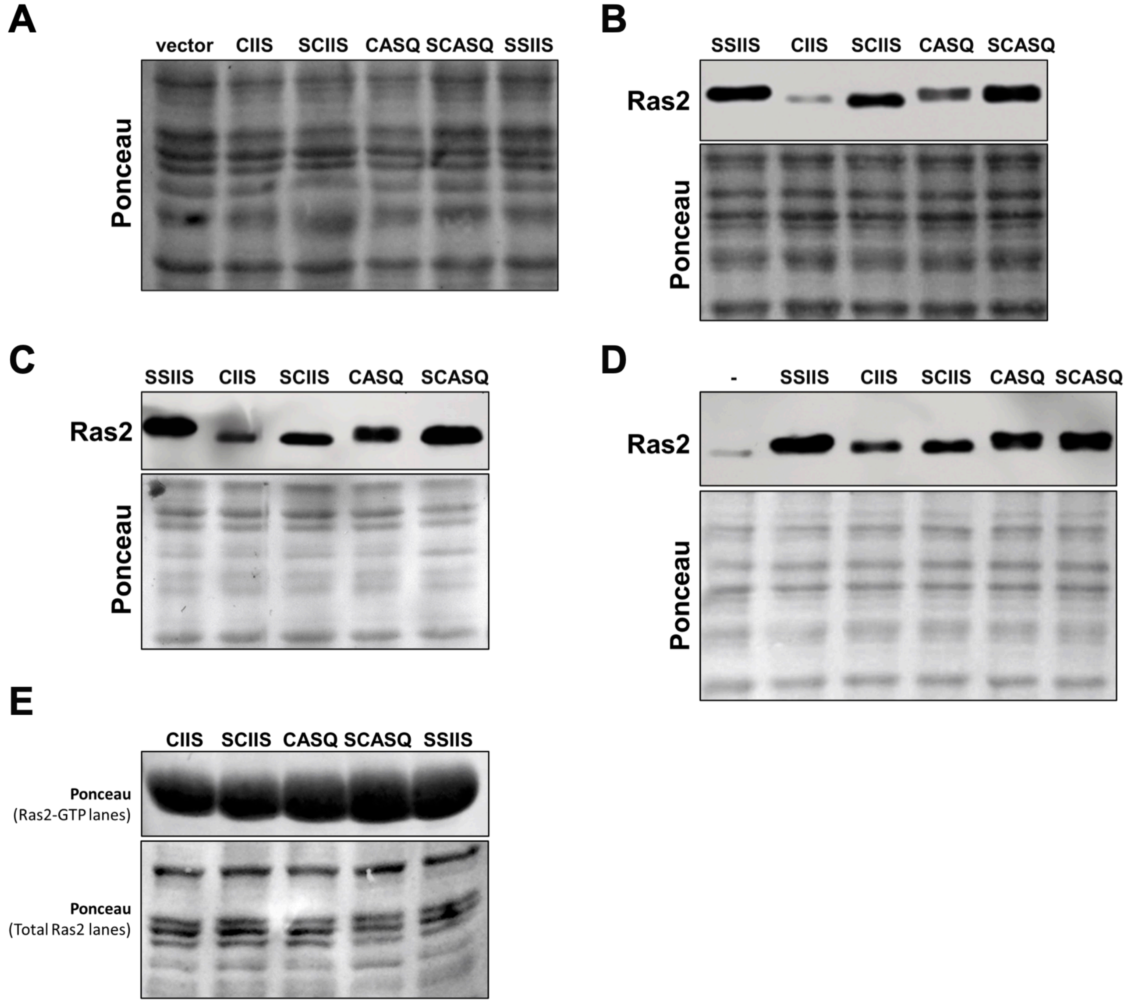

Figure S2. Impact of CAAX motifs on Ras2 protein levels. A) Ponceau S staining of blot used for **Fig. 4A** prior to antibody incubation. **B**) Anti-Ras2 immunoblot and Ponceau S staining of whole cell lysates prepared by alkaline lysis from cells grown to ~1.0 A_{600} in SC-Ura, Leu liquid media. The strain used was RJY510; plasmids used were B250, Ras2-SCaax, Ras2-SSaax, pWS1613 and pWS1615. **C**) Anti-Ras2 immunoblot and Ponceau S staining for lysates prepared by bead-beating from 5-FOA selected cells grown to ~1.0 A_{600} in SC-Leu liquid media, except for cells expressing Ras2-SSIIS that were grown in SC-Ura, Leu liquid media because they cannot be recovered after 5-FOA selection. The strain used was RJY510; plasmids used were B250, Ras2-SCaax, Ras2-SSaax, pWS1613 and pWS1615. **D**) Anti-Ras2 immunoblot and Ponceau S staining for whole cell lysates prepared by alkaline lysis from cells grown to ~1.0 A_{600} in SC-Leu liquid media. The strain used was LRB938; plasmids used were B250, Ras2-SCaax, Ras2-SSaax, pWS1613 and pWS1615. The dash (-) indicates an untransformed strain that was processed in parallel to demonstrate endogenous Ras2 levels. **E**) Ponceau S staining of blots used for **Fig. 4C** prior to antibody incubation. The major band in the Ras2-GTP blot reflects GST-Raf1-RBD that is added to capture the Ras2-GTP species.

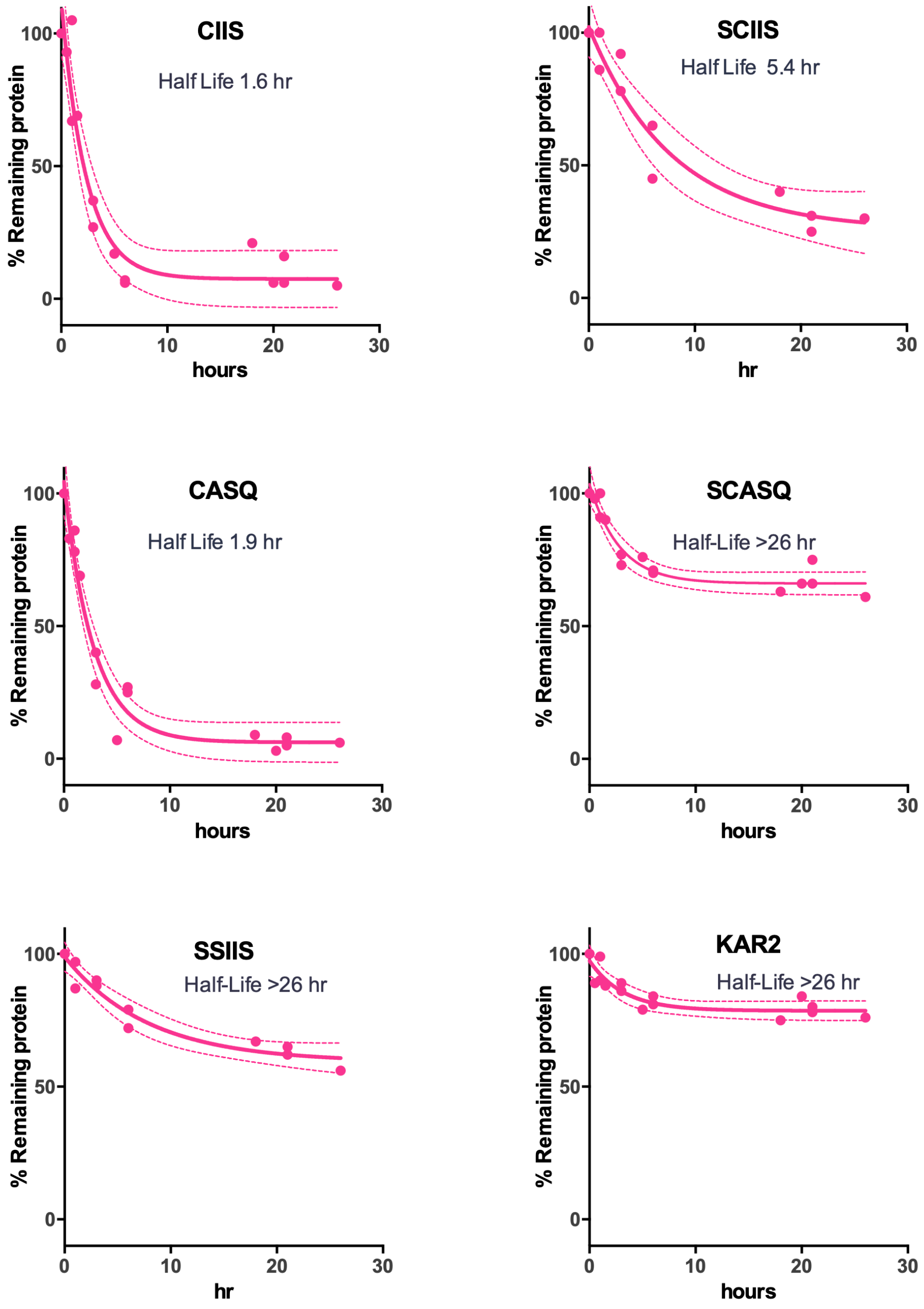

Figure S3. Evaluation of protein turnover for Ras2-CAAX variants. Band intensities for Ras2-CAAX variants and Kar2 that were reported in **Fig. 4B** were evaluated using ImageJ from appropriate immunoblot exposures. Band intensities were normalized to the respective values determined for each Ras2-CAAX variant or Kar2 at the initial timepoint (i.e., t = 0 min). The obtained values were graphed using Prism6 with curve fitting to one-phase decay. Dashed lines represent 95% confidence interval. Ras2 values were quantified using multiple biological replicates (5 for CIIS and SCASQ; 2 for SCIIS, CASQ, and SSIIS) and at least 2 immunoblot exposures from each replicate. Kar2 values were quantified using 5 biological replicates where each replicate was associated with a distinct Ras2-CAAX variant.

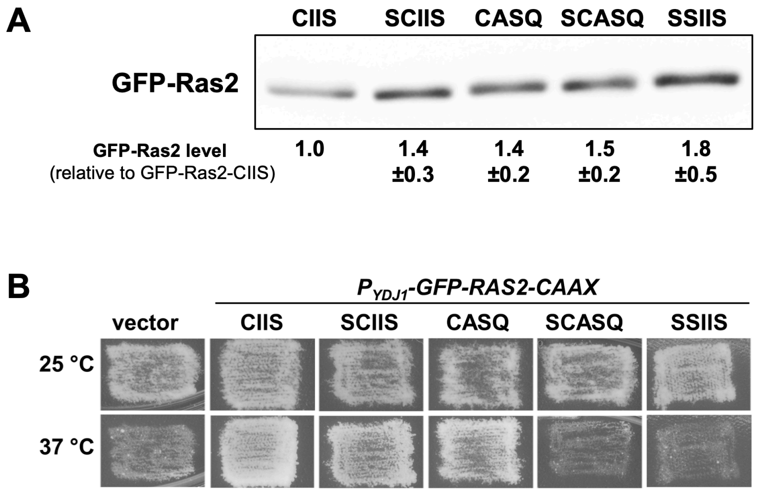

Figure S4. Impact of CAAX motifs on GFP-tagged Ras2 variants. A) Protein extracts from exponentially growing cells were prepared by alkaline lysis and analyzed by anti-GFP immunoblot. Bands were quantified using ImageJ and normalized to GFP-Ras2-CIIS. Errors represents the range from two biological replicates. Strains used were same as for **Fig. 5B**. **B**) A *ras2-23*^{ts} temperature-sensitivity assay was performed using GFP-Ras2-CAAX variants. Cells were patched on SC-Ura solid media then replica plated onto plates (shown) that were incubated at 25 °C (permissive) and 37 °C (restrictive) for 5 days. The data shown is representative of growth observed across at least 3 independent experiments having both biological and technical replicates. The strain used was JRY5318 (*ras2-23*^{ts})*;* plasmids used were pWS1501, pWS1714, pWS1735, pWS1821, pWS1823 and pWS1889.

A

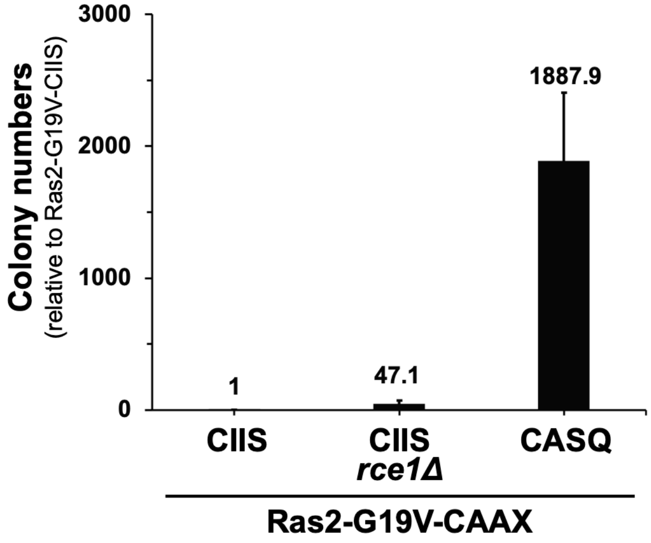

B

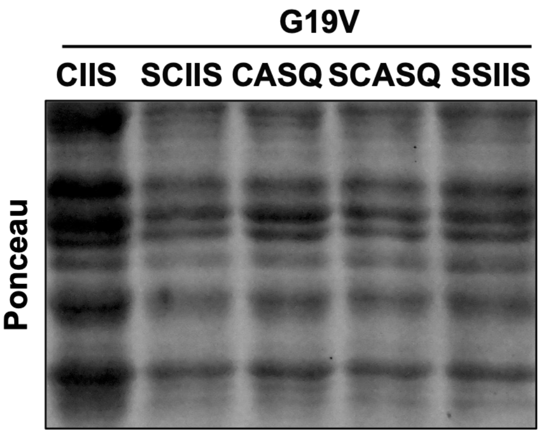

Figure S5. Quantification of acute heat-shock assay response. A) The acute heat-shock assay was performed as described for **Fig. 6A-B** and appropriate dilutions used to determine CFUs for heat-shocked samples. The values shown above each bar represent the average CFUs observed from 3 independent quantifications; error bars depict SD. The bar for Ras2-CIIS is plotted but not visible due to the scale of the graph. Yeast strains used were LRB938 and yWS3126; plasmids used were B561 and pWS1612. **B**) Ponceau S staining of blot used for **Fig. 6D** prior to antibody incubation.

Supplementary Tables:

Table S1: Oligonucleotides used for plasmid constructions in the study.

Oligo	Sequence (5´→3´)
oWS219	TGACCATGATTACGCCAAGC
oWS333	AACGAAATAAATTGATGCTTTGACTACTGACTGTCTGTCATAGAGAGAACCAGAACAGCAACATGGAGGCCCAGAATACCC
oWS334	AGTGAAAAAAGGTTATAGTACATAGGAAATTTTAAACGGTATACAAGTACGTAAAAAAGGCAGTATAGCGACCAGCATTCAC
oWS1052	TTTTCAAGAAATGAAAAGGCAATAG
oWS1096	ATGAATCGTGAATAAGTTGATCTTTTTTTATCAAGAAAATCATCTAGATTTGTATAGTTCATCC
oWS1105	AATCGTGAATAAGTTGATCTTTTTTTATCAAGAAAATCACTGTGAAGCACAACAGCCACCCGATCCGCT
oWS1109	AATCGTGAATAAGTTGATCTTTTTTTATCAAGAAAATCACTGTGAAGCACAAGAGCCACCCGATCCGCTCT
oWS1190	ACCAGTGAAGCCTCCAAGAGCGGATCGGGTGGCTGTTGTGCTTCACAGTAATAAAAAGGAAATAGTTGTAGA
oWS1191	ACCAGTGAAGCCTCCAAGAGCGGATCGGGTGGCTCATGTGCTTCACAGTAATAAAAAGGAAATAGTTGTAGA
oWS1249	AATCGTGAATAAGTTGATCTTTTTTTATCAAGAAAATCAACTTATAATACAACAGCCACCCGATCCGCTCT
oWS1250	AATCGTGAATAAGTTGATCTTTTTTTATCAAGAAAATCAACTTATAATACAAGAGCCACCCGATCCGCTCT
oWS1252	AATCGTGAATAAGTTGATCTTTTTTTATCAAGAAAATCAACTTATAATAGAAGAGCCACCCGATCCGCTCT
oWS1312	GGGATTACACATGGCATGGATGAACTATACAAATCTAGAGCATGCATGCCTTTGAACAAGTCGAAC

Table S2: Plasmid construction details.

Plasmid	Genotype	Vector (digest)	Insert (PCR components or digest)	Method[1]
pWS714	*CEN URA3 rce1::NAT*	pSM1275 (*SphI*, *HpaI*)	PCR (p4339, oWS333, oWS334)	R
pWS1501	*CEN URA3 P_{YDJ1}-GFP*	pWS1390 (*Nhe*I, *Bsa*BI, *Nar*I)	PCR (pWS1389, oWS1052, oWS1096)	R
pWS1612	*CEN LEU2 RAS2-G19V-CASQ*	B561 (*Nde*I)	PCR (B250, oWS219, oWS1190; *Psi*I, *Pst*I)	R
pWS1613	*CEN LEU2 RAS2-CASQ*	B250 (*Nde*I)	PCR (B250, oWS219, oWS1190; *Psi*I, *Pst*I)	R
pWS1614	*CEN LEU2 RAS2-G19V-SCASQ*	B561 (*Nde*I)	PCR (pWS1574, oWS219, oWS1191; *Psi*I, *Pst*I)	R
pWS1615	*CEN LEU2 RAS2-SCASQ*	B250 (*Nde*I)	PCR (pWS1574, oWS219, oWS1191; *Psi*I, *Pst*I)	R
pWS1714	*CEN URA3 P_{YDJ1}-GFP-RAS2-CASQ*	pWS1390 (*Bsa*BI, *Nar*I, *Nhe*I)	PCR (pWS1613, oWS1312, oWS1105)	R
pWS1735	*CEN URA3 P_{YDJ1}-GFP-RAS2-CCIIS*	pWS1390 (*Bsa*BI, *Nar*I, *Nhe*I)	PCR (B250, oWS1312, oWS1249)	R
pWS1821	*CEN URA3 P_{YDJ1}-GFP-RAS2-SCASQ*	pWS1390 (*Bsa*BI, *Pfl*MI, *Nhe*I)	PCR (pWS1615, oWS1312, oWS1109)	R
pWS1823	*CEN URA3 P_{YDJ1}-GFP-RAS2-SCIIS*	pWS1390 (*Bsa*BI, *Pfl*MI, *Nhe*I)	PCR (Ras2-SCaax, oWS1312, oWS1250)	R
pWS1889	*CEN URA3 P_{YDJ1}-GFP-Ras2-SSIIS*	pWS1390 (*Bsa*BI, *Pfl*MI, *Nhe*I)	PCR (Ras2-SSaax, oWS1312, oWS1252)	R
pWS1890	*CEN LEU2 RAS2-G19V-SSIIS*	Ras2-SSaax (*Sph*I, *Afe*I)	B562 (*Sph*I, *Afe*I)	L

[1]Method refers to the cloning technique used to create the plasmid: (R)ecombination, (L)igation.
